# Effect of the Characteristic Properties of Membrane on Long-Term Stability in the Vacuum Membrane Distillation Process

**DOI:** 10.3390/membranes11040252

**Published:** 2021-03-31

**Authors:** Yuki Suga, Ryousuke Takagi, Hideto Matsuyama

**Affiliations:** 1 Research Center for Membrane and Film Technology, Department of Chemical Science and Engineering, Kobe University, Nada, Kobe 657-8501, Japan; suga.yg@om.asahi-kasei.co.jp (Y.S.); takagi@harbor.kobe-u.ac.jp (R.T.); 2 AsahiKASEI Corporation, Chiyoda-ku, Tokyo 100-0006, Japan

**Keywords:** membrane distillation, hollow fiber membrane, polyvinylidene difluoride, hydrophobic treatment, long term operation, liquid entry pressure

## Abstract

Membrane distillation (MD) is a technology that can treat feed solutions with higher osmotic pressure, as well as produce high-purity water. However, the water production cost of the MD process is expensive. In this study, to decrease the water production cost, we attempted to evaluate the effect of membrane characteristics on the long-term stability of a vacuum MD (VMD) system. We fabricated four different types of polyvinylidene difluoride hollow fiber membranes, and operated a VMD system with 3.5 wt% NaCl aqueous solution at 65 °C as a feed under 11 kPa of air gap pressure. Consequently, in the proposed VMD system, it is found that the liquid entry pressure (LEP) is the most important factor. When LEP was higher than 0.37 MPa, the pilot-scale module was very stable for long-term operations, and the vapor flux was approximately 19.3 kg/m^2^·h with a total salt retention factor of over 99.9% during the 300-h operation.

## 1. Introduction

In the 21st century, water shortages are a global issue owing to population growth, as well as the associated developments of resource mining, agriculture, and industry [1]. The membrane process is one of the key technologies used to address water shortage. Presently, reverse osmosis (RO) is a mainstream process for water production, and large-scale seawater desalination plants are operated with this process [2]. The RO process is a pressure-driven membrane process in which product water is obtained by applying a higher pressure than the osmotic pressure difference between the feed and permeate to the feed side. Thus, it is difficult to treat a feed with extremely high osmotic pressure with RO. However, recently, the demand for water treatments that cannot be handled by the RO process is increasing. For these water treatments, membrane distillation (MD) processes have garnered attention as alternative technologies [3].

The MD process is a vapor pressure-driven process. In the MD process, vapor permeates through a hydrophobic porous membrane using the vapor pressure difference between feed side and permeate side as a driving force by applying a high temperature feed on one side of the hydrophobic porous membrane, and cool water on another side, while the liquid does not permeate because a hydrophobic membrane is employed [3]. Therefore, even very small non-volatile solutes such as inorganic ions are completely rejected, and very high-purity water is obtained by liquefying this vapor. In principle, MD can treat any saline solution. Even for MD systems with such high performances for water treatment, the expensive water production cost poses a significant challenge [4,5,6]. In MD process, heat energy and membrane cost are the large portions of the water production cost [6]. To reduce the heat energy cost, waste heat [7] or natural energy, such as sunlight, are considered [8]. In addition, it is important to increase the heat efficiency of the installation [3]. Heat efficiency significantly depends on the operation setup. So, it is important to select a setup with high heat efficiency. In addition, to reduce the practical membrane cost, it is important to increase the vapor flux (kg/m^2^·h) and lifetime of the MD membrane.

**Figure 1:**
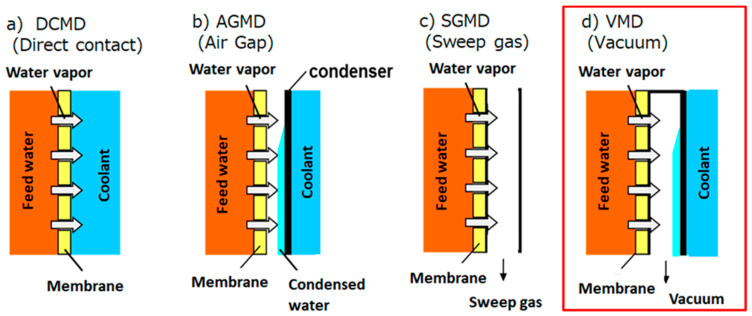
Schematics of typical MD operation setups. (**a**) DCMD, (**b**) AGMD, (**c**) SGMD, (**d**) VMD. VMD was used in this paper.

As illustrated in Figure 1, the typical MD setups are divided into four systems [9]: direct contact MD (DCMD) (Figure 1a), air gap MD (AGMD) (Figure 1b), sweep gas MD (SGMD) (Figure 1c), and vacuum MD (VMD) (Figure 1d). DCMD is the simplest MD operation method and has been reported in many papers [3]. In DCMD, feed water is in contact with a coolant via a membrane and the pass for its vapor permeation is the shortest. Therefore, the vapor flux of DCMD is very high. However, heat efficiency is not high because heat conduction through the membrane is most likely to occur [10], and the temperature polarization decreases the flux [11]. AGMD suppresses the heat conduction through the membrane by providing an air gap between the membrane and cooling section [12]. Consequently, its heat efficiency was higher than that of DCMD. However, its vapor flux is lower than that of DCMD owing to the lower vapor pressure difference. To speed up the diffusion transfer of vapor, in SGMD, sweep gas flows through the air gap part [13], while the air gap part is decompressed in the VMD [14]. Therefore, it is possible to achieve high vapor flux and low heat conduction simultaneously by SGMD and VMD. Especially, in VMD, the highest vapor flux is expected since the high vapor pressure difference can be obtained by decompressing the permeate side. In addition, in VMD, the effect of a temperature polarization will be neglected since the vapor pressure in the permeated side is determined by the decompressed pressure of the permeate side. Additionally, VMD systems can prevent feed water from contaminating the permeated water because the membrane and condenser can be placed farther apart than DCMD and AGMD. In the ideal VMD case, it is almost unnecessary to operate the vacuum pump once the air gap part is decompressed during VMD operation. Thus, the energy cost for decompressing will be low.

From the perspective of the total water production cost, VMD is considered to be the most efficient operation system. However, there are some specific problems for VMD. For example, the higher liquid entry pressure (LEP), which is defined as the pressure required for the liquid to penetrate the membrane [15], than other systems will be required since the large transmembrane pressure difference due to decompressing causes a wetting of membrane. It is also considered that the leaked salts through the membrane may crystalize on the permeate side of membrane surface. Thus, it is almost impossible to evaluate the membrane wetting (salt retention) continuously. In this study, taking these situations into consideration, we used the VMD as the operating system and we attempted to evaluate the effect of the characteristic properties of membrane on VMD performance, especially on the long-term stability of MD membrane. To achieve this goal, we fabricated four different types of polyvinylidene difluoride (PVDF) hollow fiber (HF) membranes [16,17,18] using the well-known methods. There is no novelty in the membrane fabrication methods, but the understanding on parameters which effectively affect the long-term stability of MD membrane will indicates the guiding principle of developing high performance MD membrane. Such kind of study has not been reported yet, as far as we know.

## 2. Materials and Methods

### 2.1. Materials

Solef 6010 and Solef 6020 (SOLVAY, Brussels, Belgium) were used as PVDF resins for the M-1 [16,17] and M-3 membrane fabrications [18], respectively. AEROSIL-R972 (NIPPON AEROSIL, Tokyo, Japan) was used as the hydrophobic silica which is a pore-forming agent. Di (2-ethylhexyl) phthalate (DOP), dibutyl phthalate (DBP) and glycerol triacetate (GTA) were used as diluents of the PVDF polymer. Diethyl phthalate (DEP) was used as an extruded solvent in a thermally induced phase separation (TIPS) process. CH_2_Cl_2_, EtOH, and NaOH were used to wash the membrane after fabrication. 1-Buthanol was used to measure membrane porosity. NaCl was used as a model electrolyte in the feed solution. All these chemicals were purchased from FUJIFILM Wako Pure Chemical Corporation, Osaka, Japan. A fluoropolymer FS-392B (Fluoro Technology Co. Ltd., Aichi, Japan) was used as the hydrophobic agent.

### 2.2. Fabrication of PVDF Membrane

#### 2.2.1. Fabrication of PVDF Hollow Fiber Membrane

Four different types of PVDF membranes, M-1–M-4, were fabricated in this study. First, we fabricated M-1 and M-3 membranes by TIPS method. Then, M-2 and M-4 membranes were obtained by the hydrophobic treatment of M-1 and M-3, respectively, as discussed in Section 2.2.3. The fabricating conditions for M-1 and M-3 are listed in Table 1.

M-1 is fabricated by the method described in the patent [16]. The dope solution of M-1 was a mixture of hydrophobic silica, DOP, DBP, and PVDF at a weight ratio of 23:31:6:40. This dope solution was melted at 240 °C and extruded through the outer slit of a double-orifice spinneret. Simultaneously, nitrogen gas was discharged as a hollow part formation fluid from the inner slit of the spinneret. The extruded dope was introduced into a water bath (40 °C) through a 20 cm air gap and wound up at a speed of 20 m/min. Then, the stretching process was conducted on the obtained membrane. Initially, tension was applied to the membrane to stretch it to double its length, after which the tension was released. The final membrane length was 1.5 times longer than that of the prepared membrane. Next, the membrane was immersed in CH_2_Cl_2_ to remove DOP and DBP, and then dried. Subsequently, the membrane was immersed in a 50 wt% EtOH aqueous solution and then immersed in 5 wt% NaOH aqueous solution at 40 °C for 1 h to remove silica. It was revealed that silica particles were removed completely by the analysis of the membrane composition. After washing with water and drying, the PVDF hollow fiber membrane M-1 was obtained.

M-3 was also fabricated by TIPS using the triple-orifice spinneret. M-3 was fabricated according to the method described in a previous study [18]. Briefly, GTA was used as the bore liquid and DEP as the solvent extruded through the outermost channel of the triple-orifice spinneret. The dope solution of M-3 was a mixture of GTA and PVDF at a weight ratio of 67:33.

#### 2.2.2. Preparation of Membrane Modules

**Figure 2:**
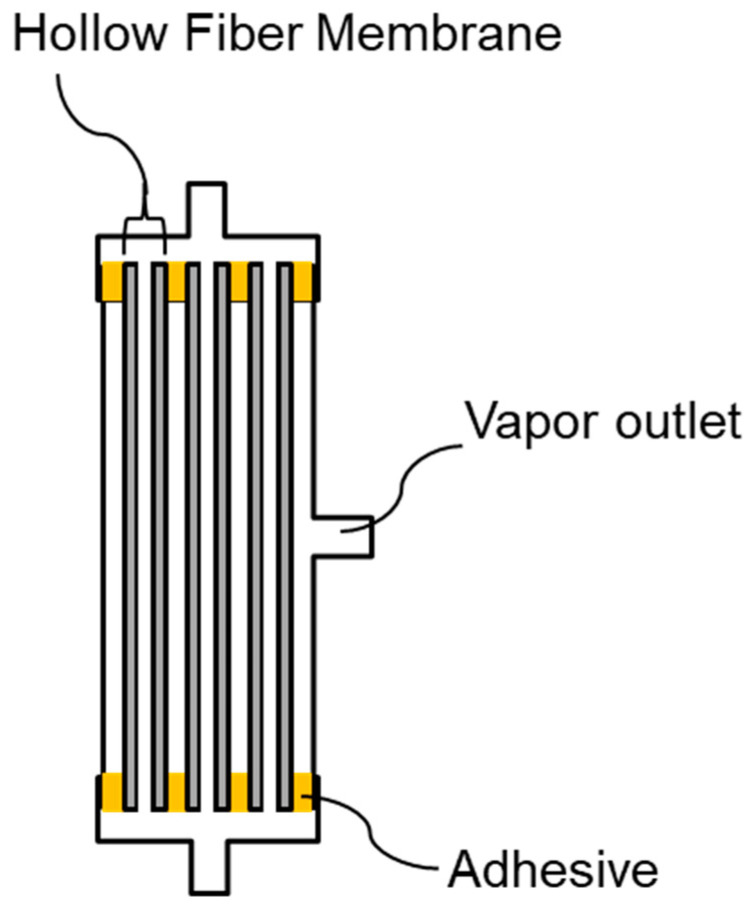
Schematic of membrane modules for VMD.

The modules are distinguished by membranes; for example, the module installed by M-1 is called the M-1 module. The M-1 lab-scale module was fabricated by inserting 35 of M-1 membranes, 11 cm in length, into a pipe and both ends were cured with a urethane adhesive. The effective bore surface area of the membrane in the lab-scale module was 0.006 m^2^ (Figure 2). The M-3 lab-scale module with the same bore surface area was made by introducing 55 of M-3 membranes, 11 cm in length, into a pipe. A pilot-scale module was fabricated in the same way as the lab-scale modules using 700 M-1 membranes with lengths of 50 cm. The effective bore surface area of the membrane in the pilot-scale module was 0.44 m^2^.

#### 2.2.3. Hydrophobic Treatment

**Figure 3:**
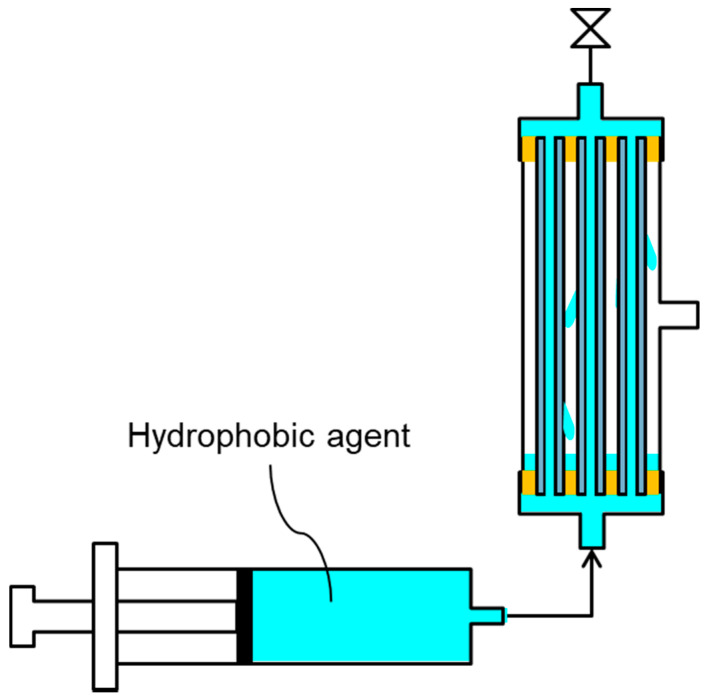
Schematic diagram of hydrophobic treatment.

One side of the M-1 lab-scale module was sealed, and then a hydrophobic agent was injected into the inside of the hollow fiber membranes from another side of the module to wet the whole membrane (Figure 3) [17]. The outer surface of the HF membranes were also wetted by a permeated hydrophobic agent (fluoropolymer FS-392B). After the entire membrane was wetted, excess hydrophobic agent was removed. Then, the membrane was dried overnight at around 25 °C by dry air flowing into the module. This operation was repeated twice and the M-2 lab-scale module was obtained. Using this operation, entire parts, including bore surface, shell surface, and cross section of the membrane, were hydrophobized. The same hydrophobic treatment was performed on the M-3 lab-scale and M-1 pilot-scale modules to fabricate the M-4 lab-scale and M-2 pilot-scale modules, respectively. 

### 2.3. Characterization of PVDF Membrane

#### 2.3.1. Pore Size Distribution and Porosity

The pore size distributions of all the hollow fiber membranes were measured using a liquid-liquid porometer (LLP-1100A, Porous Materials, Inc., Ithaca, NY, USA) [18]. In this method, pore presence was detected by sensing an increase in the flow rate at a given applied differential pressure, after which “mean flow pore size” was calculated.

The porosity of each hollow fiber membrane was measured via the gravimetric method [19].

#### 2.3.2. LEP Measurement

**Figure 4:**
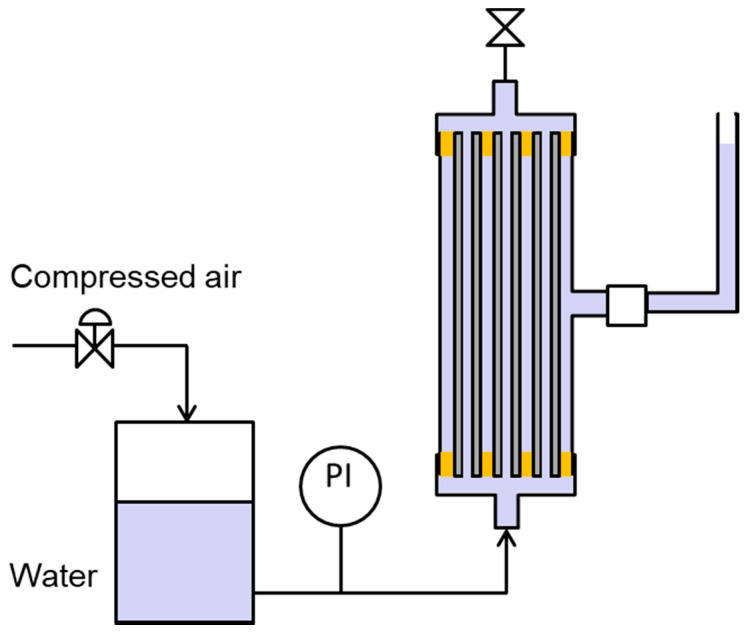
How to measure LEP of membrane.

To measure the liquid entry pressure (LEP) of each membrane, both the bore and shell sides of the membrane installed in the lab-scale module were filled with water, and then pressure was applied to the bore side (Figure 4). Pressure was gradually increased while observing the water level in the tube attached to the shell outlet of the module. LEP was determined as the pressure at which the water level in the tube began to rise [20].

#### 2.3.3. Polymer Composition of Membrane Surface

To confirm the change of surface condition before and after hydrophobic treatment, polymer composition of M-1 and M-2 were observed by XPS (ESCALAB250, Thermo Fisher Scientific, Inc., Waltham, MA, USA). AlKα (15 kV × 10 mA) was used as the X-ray source. Membrane sample was prepared by cutting into around 1 mm size and open the hollow fiber to be able to analyze bore surface. Bore surface of M-1 and M-2 were analyzed, and shell surface of M-2 was also analyzed. The presence of fluoropolymers derived from hydrophobic agent was identified by comparing the peak at 292 eV which is considered to be derived from a fluoroethylene carbon of the hydrophobic agent.

#### 2.3.4. VMD Evaluation

In the evaluation of the MD performance, especially long-term operation, an increasing feed concentration greatly affects the result, because of the change of water activity coefficient in the feed. Furthermore, if precipitation of the salt occurs with condensation of feed, it may cause a clogging of membrane pore resulting in a decreasing flux. Thus, in this study, the feed concentration was kept constant to avoid such effects, and to evaluate the effect of characteristic property of membrane accurately.

**Figure 5:**
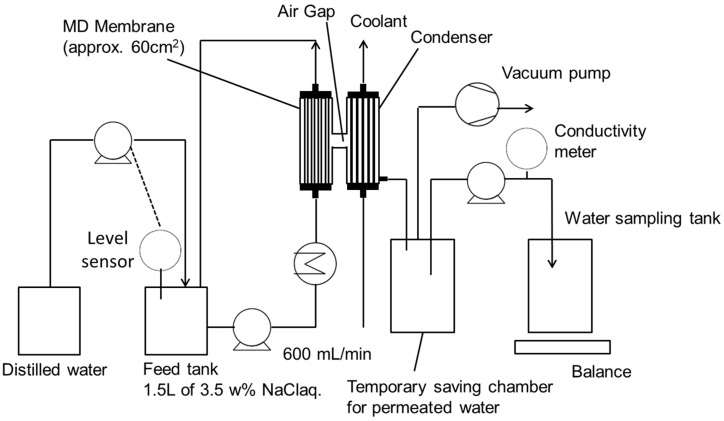
Schematic of lab-scale VMD system.

The evaluation of the MD performance of the lab-scale module was conducted using the equipment shown in Figure 5. A 3.5 wt% NaCl aqueous solution was used as the feed and heated to 65 °C, then it was circulated to the bore side of the membrane module at a flow rate of 600 mL/min. When the feed volume was reduced via the MD operation, a liquid level sensor installed in the feed tank switched on the pump to supply distilled water and maintain a constant NaCl concentration. The condenser connected to the membrane module was cooled below 20 °C by circulating cooling water at a flow rate of 1000 mL/min. The condenser was also connected to a temporary saving chamber for the permeated water. The shell side of the membrane module, inside the condenser, and saving chamber were maintained at a pressure of 11 kPa using a vacuum pump. The permeate water stored in the temporary saving chamber was discharged into the water sampling tank, while the salt concentration of the permeated water was measured by the in-line conductivity meter. The vapor flux through the membrane is given by Equation (1):

(1) $$J_{w}=\;\frac{W_{p}}{A\cdot T}$$

where *J_w_* (kg/m^2^·h), *W_p_* (kg), *A* (m^2^), and *T* (h) represent water vapor flux, weight of the permeated water, effective membrane bore surface area, and operating time, respectively [21].

Leaking salt flux (g/m^2^·h) was obtained from the operating time and weight of salt permeated through the membrane. Generally, VMD membrane performances were solely evaluated by permeated water quality [21,22,23,24,25]. However, during VMD operation, leaking salt exists not only in permeated water alone but also on the membrane shell surface. In this study, to evaluate membrane performance more accurately, we washed the shell side of the membrane module after the VMD operation to determine the amount of salt on the shell surface of the membrane. Then, the total amount of leaking salt was calculated using Equations (2)–(4),

(2) $$J_{st}\;=\;J_{sp}\;+\;J_{sr}$$

(3) $$J_{sp}\;=\;\frac{1000m_{p}}{A\;T}=\;\frac{1000W_{p}C_{p}}{A\;T}$$

(4) $$J_{sr}=\;\frac{1000m_{r}}{A\;T}=\;\frac{1000W_{w}C_{w}}{A\;T}$$

where *J_st_* (g/m^2^·h), *J_sp_* (g/m^2^·h), and *J_sr_* (g/m^2^·h) represent the total leakage salt flux, leaking salt flux into permeated water, and leaking salt flux remaining on the shell surface of the membrane, respectively. *J_sp_* and *J_sr_* are given by Equations (3) and (4), respectively, where *m_p_* (kg), *C_p_* (wt%), *m_r_* (kg), *W_w_* (kg), and *C_w_* (wt%) represent the weight of salt in permeated water, salt concentration in permeated water, weight of the salt remaining on the shell surface of the membrane, weight of washing water, and salt concentration in the washing water, respectively. Additionally, *C_p_* and *C_w_* were obtained from the electrical conductivity of the permeated water and washing water, respectively.

The salt retention factor, *r_F_* (%), is calculated using Equations (5)–(7),

(5) $$r_{F}=\;\left\{1\;-\;\frac{C_{p}^{0}}{C_{f}}\right\}\;\times \;100$$

(6) $$C_{p}^{0}=\;\frac{m_{p}^{0}}{W_{p}}\;\times 100\;$$

(7) $$m_{p}^{0}\;=\;m_{p}+m_{r}$$

where *C_f_* (wt%), *C_p_^0^* (wt%), and *m_p_^0^* (kg) represent the salt concentration in the feed, accurate salt concentration of permeated water given by Equation (6), and weight of the totally permeated salt given by Equation (7), respectively. 

**Figure 6:**
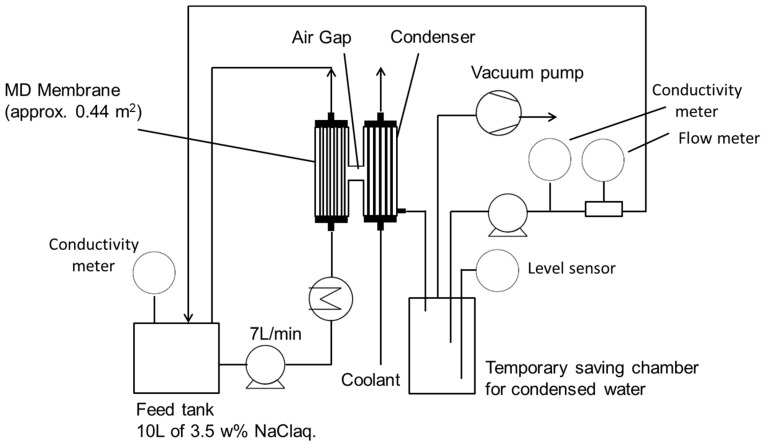
Schematic of pilot-scale module. The salt concentration of feed water was kept constant by returning the permeated water to the feed tank.

The evaluation of the M-2 membrane pilot-scale module was conducted using the equipment illustrated in Figure 6. Here, 10 L of 3.5 wt% NaCl aqueous solution was used as the feed. The feed was heated to 65 °C and supplied to the bore side of the M-2 membrane at a flow rate of 7 L/min. The pressure in the condenser was maintained at 11 kPa by a vacuum pump. Tap water (<40 °C) was used as the coolant and supplied to the condenser at a flow rate of 10 L/min. The permeate was stored in the temporary saving chamber once. The chamber had a level sensor to monitor the level of the permeate in the camber. When the certain amount (around 3 L) of the permeate accumulated in the chamber, the level sensor turned on the permeate discharging pump to return the permeate to the feed tank until the level reaches below lower limit of the sensor to maintain the constant salt concentration of the feed. The volume of accumulated permeated water and its salt concentration were measured by an integrated flow meter installed at the permeated water discharging pump, and a conductivity meter, respectively, when the permeate was returned to the feed tank. Flux was obtained from the operating time and the volume of permeated water. The weight of permeated water was calculated using its density as 1.0 g/mL because the permeated water was almost pure, as mentioned later. The total amount of leaking salt was calculated as a sum of the amount of salt contained in the permeated water and the amount of salt remaining on the shell surface of the membrane. These were obtained similar to that of the lab-scale VMD evaluation.

## 3. Results and Discussion

### 3.1. Membrane Morphology

**Figure 7:**
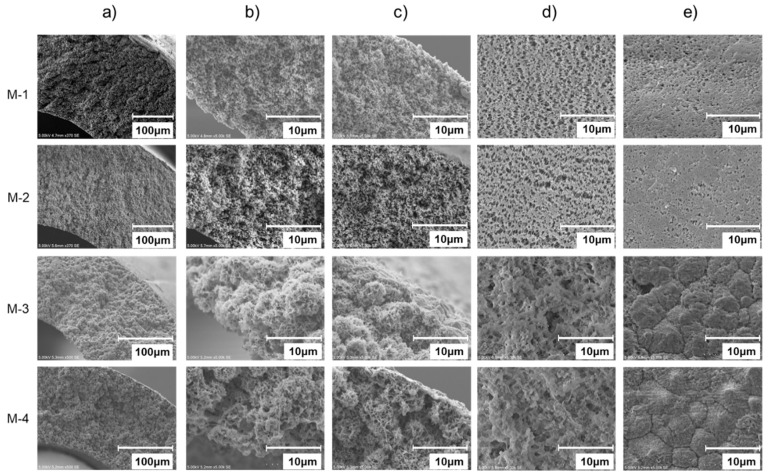
SEM images of M-1—M-4 membrane. (**a**) Cross section, (**b**) Near the bore side of the cross section, (**c**) Near the shell side of the cross section, (**d**) Bore surface, (**e**) Shell surface.

**Figure 8:**
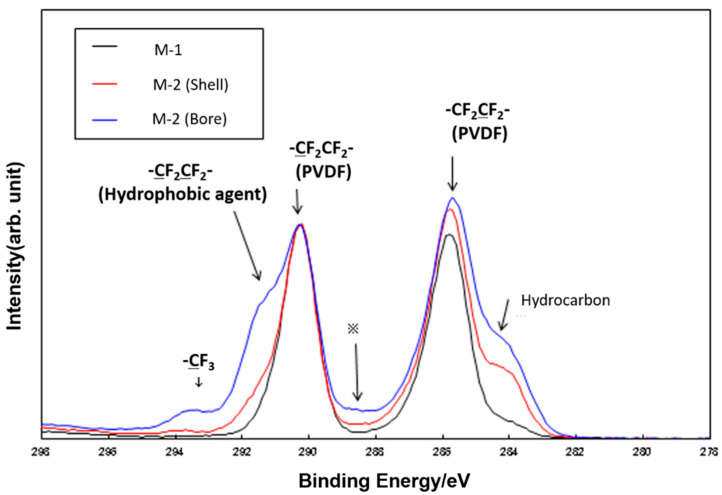
The results of XPS analysis of M-1 and M-2.

Figure 7 shows SEM images of M-1–M-4. It is found from Figure 7 that M-1 has a highly porous and uniform sponge-like structure throughout its cross section. Its bore surface porosity is higher than that of the shell surface. In contrast, M-3 consists of spherulites with a diameter of approximately 10 μm. There are several micron pores on the bore surface. Although the number of pores on the shell surface is less than that on the bore surface, there are crack-like gaps between spherulites. Comparing SEM images of M-1 with M-3, the pore size of M-1 appears to be more uniform than that of M-3, and the porosity on the bore surface of M-1 seems to be higher than that of M-3. This is due to the silica particles added as a pore forming agent in fabricating M-1. Additionally, from these SEM images, it can be also seen that silica does not remain in the M-1 and M-2 [16]. Regarding the difference of membrane morphology of M-1 and M-3, it is mainly due to the difference of the bore fluid, and existence of the extent solvent. In M-1 fabrication, the concentration of PVDF polymer arise rapidly by evaporation of the diluent as it passes through the air gap, resulting in small pore [18]. On the other hand, in M-3 fabrication, the extent solvent prevents evaporation of diluent, and the PVDF concentration becomes lower than M-1, resulting in larger pore than that of M-1. M-2 and M-4 were obtained by the hydrophobic treatment of M-1 and M-3, respectively. There is no clear difference in the SEM images between M-1 and M-2, and between M-3 and M-4. Therefore, the hydrophobic agent apparently formed a very thin layer on the polymer surface of the hollow fiber. Figure 8 shows the results of XPS analysis of the surface of M-1 and M-2. It is found that a peak at 292 eV which is considered to be derived from a fluoroethylene carbon of the hydrophobic agent is observed only on the bore and shell surfaces of M-2, although the intensity from bore surface is higher than that from shell surface. 

### 3.2. Evaluation of Physical Properties of Membranes

**Figure 9:**
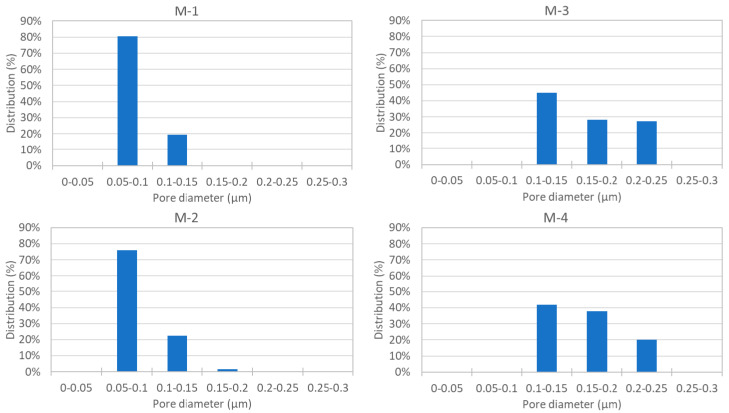
Pore size distribution of each membrane, M-1–M-4.

Table 2 presents the properties of each membrane. The outer and inner diameters of M-1 are 1.22 and 0.66 mm, respectively, and its membrane thickness is 0.28 mm. In comparison, the outer and inner diameters of M-3 are 0.75 and 0. 47 mm, respectively, and its membrane thickness is 0.14 mm. The mean pore size of M-1 is 0.10 μm, which is smaller than that of M-3 (0.19 μm). The porosity of M-1 is 72% and higher than that of M-3 (49%). The hydrophobic silica used in the membrane fabrication as a pore-forming agent may contribute to the fabrication of such a highly porous membrane [16]. The maximum pore size of M-1 is 0.12 μm, which is smaller than that of M-3 (0.23 μm). Consequently, the LEP of M-1 is 0.25 MPa, and higher than that of M-3 (0.17 MPa). Figure 9 shows the pore size distribution of M-1–M-4. In comparison, the pore size distribution of M-1 is narrower than that of M-3. In summary, from the perspective of obtaining the flux, M-1 has both an advantage (higher porosity) and disadvantages (thicker membrane and smaller mean pore size) when compared with M-3. In contrast, from the perspective of salt retention in the MD process, M-1 is superior to M-3 because M-1 has a higher LEP because its maximum pore size is smaller than that of M-3. 

When compared with M-2 fabricated by the hydrophobic treatment of M-1 [17], there were no changes in the outer diameter, inner diameter, and thickness of M-1. Furthermore, other properties such as pore size and porosity are also approximately the same. Only the contact angle of M-2 was increased from 103° for M-1 to 132° by the hydrophobic treatment, as presented in Table 2. Therefore, the LEP of M-2 also improved from 0.25 to 0.37 MPa. As mentioned above, there is very thin layer of the hydrophobic agent on the shell surface of M-2, revealed from the XPS analysis. This improvement of contact angle of the shell surface of the membrane indicates that hydrophobic treatment has remarkable effect on the hydrophobicity of the membrane surface even by the very thin layer of the hydrophobic agent.

Regarding the M-3 and M-4 fabricated by the hydrophobic treatment of M-3, both the contact angle and LEP of M-4 were also increased by the hydrophobic treatment without any change in other properties. From these results, it is expected that M-1 and M-2 exhibit the same vapor flux and deferent salt retention. Similarly, M-3 and M-4 are expected to have the same flux and deferent salt retention. By comparing these membranes, it is possible to discuss the effect of each physical property on stability during long-term VMD operation.

### 3.3. VMD Performance

#### 3.3.1. VMD Performance of Lab-Scale Module

M-1, M-2, M-3, and M-4 lab-scale modules were evaluated to confirm the relationship between vacuum MD (VMD) performance, membrane morphology, and their physical properties such as porosity, pore size distribution, and LEP. In this evaluation, 3.5 wt% of NaCl aqueous solution was used as the feed, and the lab-scale module with a 0.006-m^2^ effective bore surface area (Figure 3) was used. The VMD operation of all four modules was performed for 100 h because M-3 was wetted over 100 h, making it impossible to evaluate the performance. Table 3 presents the results of the VMD test. In the column of water vapor flux, “Initial” represents the flux during 1 h after starting operation, “Last” represents the flux from 99 to 100 h, and “Average” indicates the flux during the entire operating terms of 100 h. The leaking salt flux was obtained at the end of the operation. In the column of leaking salt flux, “Salt in permeated water” indicates the leaking salt flux calculated from the amount of salt in permeated water, “Salt remaining on membrane” represents the flux calculated from the amount of salt retained on the shell surface of the membrane, and “Total” represents the sum of “Salt in permeated water” and “Salt remaining on membrane.” “Leaking salt flux” represents the flux during a 100-h operation. The retention factor was obtained at the end of the operating term.

From Table 3, the order of the initial flux was determined to be M-2 ≥ M-1 >> M-3 ≥ M-4. As earlier mentioned, compared to M-3, M-1 has both advantages and disadvantages with respect to vapor flux. However, it is clear that M-1 exhibits a higher initial vapor flux than M-3. This was possibly because a higher porosity had a larger impact on the initial vapor flux than membrane thickness in this experiment. For the stability of the flux, the order of the last flux/initial flux is given as M-2 > M-1 >> M-4 > M-3. This order correlates with the order of LEP presented in Table 2. This indicates that the membrane with higher LEP, which is mainly determined by higher hydrophobicity and/or smaller maximum pore size [15], is stably operated for a longer time. 

**Figure 10:**
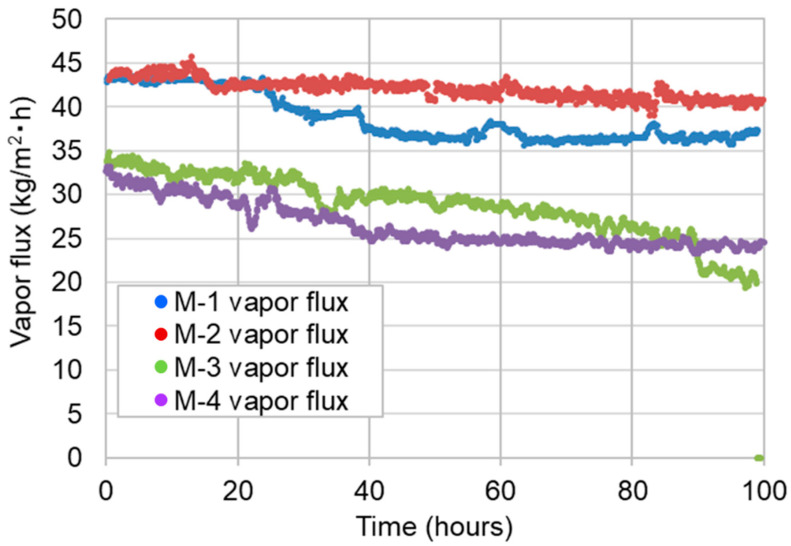
Time course of vapor flux of four membranes.

Figure 10 shows the time course of the vapor flux of the four membranes over the entire operation period of 100 h. It is clear that the vapor flux of M-2 was the most stable among the four membranes during the 100-h VMD operation. The fluxes of M-1 and M-4 gradually decreased in the first 40 h, and then became constant. The flux of M-3 continued to decrease during the 100-h operation. These results suggest that some pores with low LEP in the M-3 membrane became wet, which led to a pore clogging due to leaked salts and then, a decrease in vapor flux.

**Figure 11:**
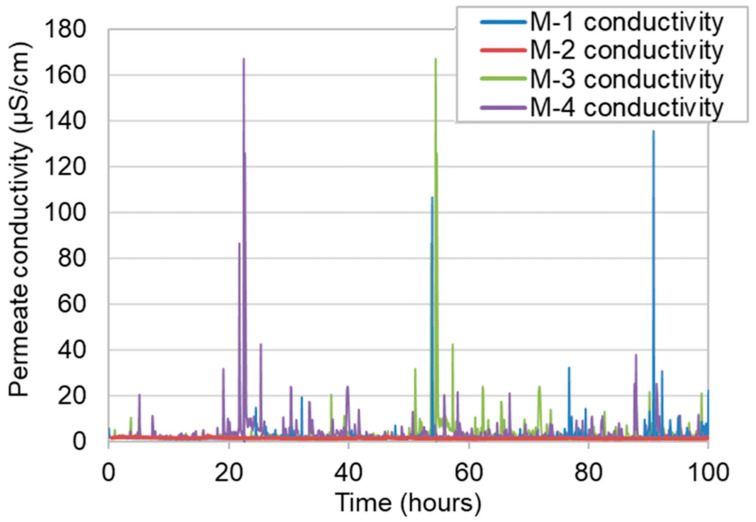
Time course of permeate water conductivity of four membranes.

**Figure 12:**
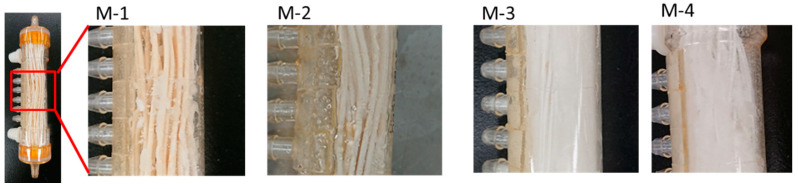
The pictures of the M-1 to M-4 lab-scale modules after 100-h VMD operation.

Figure 11 shows the time course of the permeate water conductivity of each membrane over the entire operation period. During the 100-h of operation M-1, M-3, and M-4 exhibited “spikes” in their conductivity, while M-2 did not. Figure 12 shows the shell surface after 100 h of VMD operation. From Figure 12, it is evident that in M-1, M-3, and M-4, the shell surface of each membrane was significantly or less significantly covered by the salt after the end of the operation, whereas the shell surface of M-2 was scarcely covered with salts. Therefore, it is reasonable to consider that a part of accumulated salt on the shell surface flakes from the surface occasionally, and melts in the permeate resulting in the spike in permeate conductivity. The accumulation of salt on the shell surface will also be the cause of flux decline. This indicates that the larger the LEP, the smaller the leaking salt flux. Interestingly, the salt amounts in permeated water of all membranes were almost the same, despite the significant differences in LEP, as shown in Table 3.

**Figure 13:**
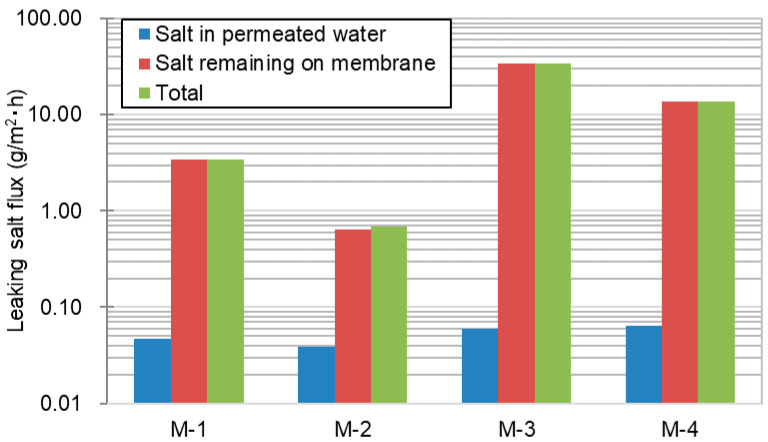
Comparison of leaking salt flux of four membrane modules during the 100-h operation.

Figure 13 shows three kinds of leaking salt flux of four lab-scale membrane modules during the 100-h operation that were calculated from the permeated water conductivity, amount of salt attached on the shell surface of membrane, and total leaking salt flux (their total sum), respectively. The order for the total leaking salt flux is M-3 (34.39 g/m^2^·h) > M-4 (13.67 g/m^2^·h) > M-1 (3.44 g/m^2^·h) > M-2 (0.68 g/m^2^·h). This order is opposite that of the LEP. Therefore, it is evident that the higher the LEP, the lower the salt leakage. Interestingly, from Figure 13, it is determined that most of the salt that permeated through the membrane remained on the shell surface of the membrane. Even in the M-3 and M-4 cases, the proportion of remaining salt on the shell surface were over 99% of the entire amount of permeated salt, as shown in Table 3. Provided it is calculated solely with the salt in permeated water, 96.6% of the retention factor of M-3 increases to over 99.9%. In other words, it is one of merits of VMD that the conductivity of permeated water does not increase so much even with such a severe membrane wetting.

**Figure 14:**
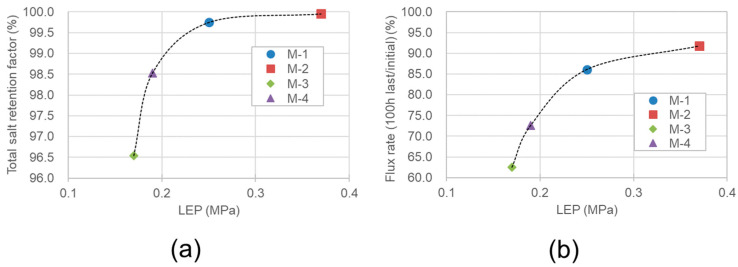
The LEP dependency of (**a**) the total salt retention factor and (**b**) the stability of water vapor flux of M-1–M-4 after the 100-h VMD operation.

Figure 14a,b present the salt retention factor and last/initial ratio of water vapor flux as a function of LEP, respectively, during the 100-h VMD operation. Owing to insufficient data, it is challenging to quantitatively discuss the relationship between LEP and salt retention, and between LEP and the last/initial ratio of water vapor flux. Nevertheless, it is evident from Figure 14a,b that the higher the LEP, the higher the salt retention factor and vapor flux stability. In the VMD system reported here, it is found that if the LEP is higher than approximately 0.32 MPa, the decline in the vapor flux is less than 10%, and the total salt retention factor is over 99.8% during the 100-h VMD operation.

#### 3.3.2. Scale-up and Long-Term Operation

**Figure 15:**
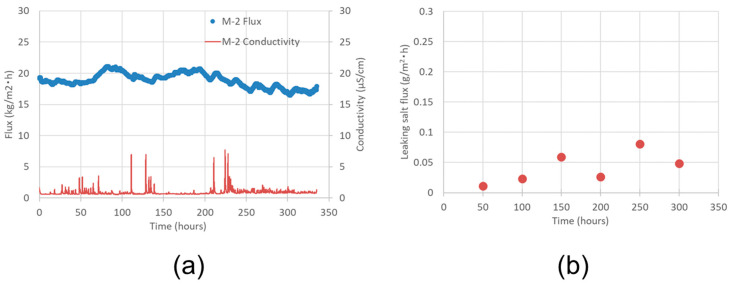
Time course of (**a**) the vapor flux and the conductivity of permeate and (**b**) the total leaking salt flux determined from the sum of salt contents in the permeated water and on the shell surface during VMD operation of M-2 pilot-scale module.

Figure 15a shows the results of the VMD long-term operation performed with the M-2 pilot-scale module. The operation conditions were the same as those for the lab-scale module, except for the feed flow rate of 7 L/min. The initial vapor flux was 19.3 kg/ m^2^·h.

After 300 h of operation, the flux was 16.8 kg/m^2^·h, which maintained 87% of the initial flux. The conductivity of the permeated water was 8 μS/cm or less throughout the entire operation period, which corresponds to less than 6.4 ppm of the total NaCl concentration of the permeated water. The total leaking salt flux, which was obtained from the sum of salt contents in the permeated water and on the shell surface, was less than 0.1 g/m^2^·h, as shown in Figure 15b. These results suggest that the M-2 pilot-scale module is very stable for long-term operations.

## 4. Conclusions

The membrane distillation (MD) process has garnered attention as a technology that can treat feed solutions with high osmotic pressures that the RO process cannot treat. In addition, MD can produce high-purity water. However, the MD process is limited by its high water production cost. The increase in the lifetime of the MD membrane corresponds to a decrease in the water production cost of MD. In this study, we attempted to evaluate membrane properties that affect the long-term stability of membranes using a VMD system. First, we fabricated two different types of PVDF hollow fiber membranes, M-1 and M-3, then obtained M-2 and M-4 by treating M-1 and M-3 with hydrophobic agents, respectively. Regarding salt retention, we evaluated the salt retention factor using both the salt in the permeate, and also the salt retained on the shell surface of the membrane. Consequently, it was evident that the higher the LEP, the higher the salt retention and vapor flux stability. Theoretically, it should be possible to operate MD without wetting if the LEP of the membrane is higher than the pressure difference between the feed and permeate. However, practically, a much higher LEP than the practical pressure difference was necessary to operate the VMD stably. In the VMD system reported here, in which the vapor pressure difference was approximately 0.1 MPa, it was found that in the case where the LEP was higher than approximately 0.32 MPa, the decrease in vapor flux was less than 10% and the total salt retention factor was over 99.8% during the 100-h VMD operation. Furthermore, we attempted the 300-h VMD operation using a pilot-scale MD system, which installed the membrane with an LEP of 0.37 MPa (M-2), and demonstrated that the M-2 pilot-scale module was very stable for long-term operations.

## Figures and Tables

**Table 1:** Fabricating conditions for PVDF hollow fiber membrane M-1 and M-3.

Spinning Conditions	M-1	M-3
Dope solution	PVDF/Si/DOP/DBP = 23/31/6/40	PVDF/GTA = 33/67
Spinneret	Double-orifice	Triple-orifice
Melting Temp. (°C)	240	190
Bore fluid	N_2_	GTA
Extruded solvent	None	DEP
Length of air gap (cm)	20	5
Take-up speed (m/min)	20	20
Elongation rate	150%	None

**Table 2:** Membrane properties of M-1–M-4.

Membrane	OD ^1^	ID ^2^	Thickness	Mean Pore Size	Maximum Pore Size ^3^	Porosity	Contact Angle	LEP
	[mm]	[mm]	[mm]	[μm]	[μm]	[%]	[°]	[MPa]
M-1	1.22	0.66	0.28	0.10	0.12	72	103	0.25
M-2	1.22	0.66	0.28	0.10	0.14	72	132	0.37
M-3	0.75	0.47	0.14	0.19	0.23	49	113	0.17
M-4	0.75	0.47	0.14	0.20	0.22	54	134	0.19

^1^ Outer diameter, ^2^ Inner diameter, ^3^ Mean flow pore size.

**Table 3:** VMD operation results for M-1–M-4 lab-scale module.

Membrane	Water Vapor Flux				Leaking Salt Flux			Retention Factor ^6^
	Initial ^1^	Last ^2^	Last/Initial	Average ^3^	Salt in Permeated Water ^4^	Salt Remaining on Membrane ^5^	Total	
	[kg/m^2^·h]	[kg/m^2^·h]	%	[kg/m^2^·h]	[g/m^2^·h]	[g/m^2^·h]	[g/m^2^·h]	%
M-1	43.2	37.2	86%	38.7	0.05	3.39	3.44	99.7%
M-2	43.9	40.3	92%	42.0	0.04	0.64	0.68	>99.9%
M-3	33.7	21.0	62%	28.8	0.06	34.33	34.39	96.6%
M-4	33.2	24.1	73%	26.5	0.06	13.60	13.67	98.5%

^1^ Obtained from the weight of permeated water during one hour after starting operation. ^2^ Obtained from the weight of permeated water during one hour before ending operation. ^3^ The flux during whole operating terms. ^4^ Obtained from the amount of salt in permeated water during whole operating term. (Equation (3)). ^5^ Obtained from the amount of salt remaining on membrane shell surface during whole operating term and operation time. (Equation (4)). ^6^ Obtained from the weight of salt in the feed and the total amount of permeated salt at the end of operation. (Equation (5)).

## Nomenclature

Symbols	Description	Units
*A*	Total membrane bore surface area	m^2^
*C_f_*	Salt concentration in feed water	wt%
*C_p_*	Salt concentration in permeated water	wt%
*C_p_^0^*	Accurate salt concentration of permeated water	wt%
*C_w_*	Salt concentration in washing water	wt%
*J_sp_*	Leaking salt flux into permeated water	g/m^2^·h
*J_sr_*	Leaking salt flux remaining on shell surface of membrane	g/m^2^·h
*J_st_*	Total leaking salt flux	g/m^2^·h
*J_w_*	Water vapor flux	kg/m^2^·h
*m_p_*	Weight of salt in permeated water	kg
*m_r_*	Weight of salt remaining on shell surface of membrane	kg
*m_p_^0^*	Weight of total permeated salt	kg
*r_F_*	Salt retention factor	%
*T*	Operating time	H
*W_p_*	Weight of permeated water	kg
*W_w_*	Weight of washing water	kg
